# Developing Successful Breeding Programs for New Zealand Aquaculture: A Perspective on Progress and Future Genomic Opportunities

**DOI:** 10.3389/fgene.2019.00027

**Published:** 2019-02-01

**Authors:** Jane E. Symonds, Shannon M. Clarke, Nick King, Seumas P. Walker, Brian Blanchard, David Sutherland, Rodney Roberts, Mark A. Preece, Mike Tate, Peter Buxton, Ken G. Dodds

**Affiliations:** ^1^Cawthron Institute, Nelson, New Zealand; ^2^AgResearch, Invermay Agricultural Centre, Mosgiel, New Zealand; ^3^Mount Cook Alpine Salmon, Christchurch, New Zealand; ^4^SPATnz, Nelson, New Zealand; ^5^The New Zealand King Salmon Co., Ltd., Picton, New Zealand; ^6^Sanford Limited, Kaitangata, New Zealand

**Keywords:** aquaculture, selection, genomics, industry benefits, king salmon, mussels

## Abstract

Over the past 40 years New Zealand (NZ) aquaculture has grown into a significant primary industry. Tonnage is small on a global scale, but the industry has built an international reputation for the supply of high quality seafood to many overseas markets. Since the early 1990s the industry has recognized the potential gains from selective breeding and the challenge has been to develop programs that can overcome biological obstacles (such as larval rearing and mortality) and operate cost-effectively on a relatively small scale while still providing significant gains in multiple traits of economic value. This paper provides an overview of the current status, and a perspective on genomic technology implementation, for the family based genetic improvement programs established for the two main species farmed in NZ: Chinook (king) salmon (*Oncorhynchus tshawytscha*) and Greenshell^TM^ mussel (*Perna canaliculus*). These programs have provided significant benefit to the industry in which we are now developing genomic resources based on genotyping-by-sequencing to complement the breeding programs, enable evaluation of the genetic diversity and identify the potential benefits of genomic selection. This represents an opportunity to increase genetic gain and more effectively utilize the potential for within family selection.

## Introduction

The New Zealand (NZ) aquaculture industry began more than half a century ago, but it was not until the 1990s that selective breeding programs were implemented for stock improvement (reviewed by Symonds et al., 2018). NZ recognizes aquaculture as an important and expanding primary industry. Currently, Greenshell^TM^ mussels (GSM) (*Perna canaliculus*) and Chinook (king) salmon (*Oncorhynchus tshawytscha*), produce over NZD $400 million (m) p.a. in export revenue; in 2017 revenue was $308 m for GSM and $97 m for king salmon ^1^. Although the NZ tonnage is small on a global scale, the industry has built an international reputation for the supply of high quality seafood to many overseas markets. Demand for NZ farmed seafood currently outstrips production and the industry has a goal to produce higher value branded products to meet domestic and export market demands. In addition to the potential of aquaculture to contribute significantly as adequate nutrition for a growing global population, there is an increasing demand for high quality seafood from an expanding middle class, as countries like China continue to develop.

Since the early 1990s the industry has recognized the potential gains from selective breeding and the challenge has been to develop programs that can overcome biological obstacles (such as larval mortalities) and operate cost-effectively on a relatively small scale while still providing significant gains. The first king salmon family breeding program was established in 1994 by commercial salmon farming company Southern Ocean Seafood Ltd. In 2002, the Cawthron Institute set up the first GSM families using wild parents and have since established a family based breeding program, now operated and managed by Breedco Ltd. In 2007, the second largest salmon farming company, Sanford Ltd., moved away from mass selection and has since developed a combined between- and within-family selection program. The breeding programs for both these species are designed so that the families are evaluated on one or more commercial farms, either in mixed family groups or as separate replicated families. To complement these well-established breeding programs and enhance emerging programs, we are now developing genomic solutions for the NZ aquaculture industry. The reduction in DNA sequencing costs coupled with multiplex assays has enabled restriction enzyme based sequencing techniques to be explored as a simultaneous SNP discovery and genotyping method for the industry. This genotyping strategy, together with advanced statistical analysis, allow genetic diversity to be assessed, pedigree established/verified and genomic selection to be implemented.

This paper provides an overview and describes the benefits to the industry from the genetic improvement programs. In addition, a perspective of the use of genotyping-by-sequencing (GBS) as a tool to deliver genomic solutions to established and emerging breeding programs is discussed. To ensure NZ aquaculture meets the desired revenue target, progress in all sectors will be required, including the implementation of practical methods of genetic improvement.

## NZ King Salmon Industry

The NZ king salmon industry is the largest producer of farmed king salmon globally, with total production currently ∼12,000 to 14,000 metric tons p.a ^2^. This Pacific salmon species was imported to NZ in the early 1900’s from the McLeod River, a tributary of the Sacramento River in California (McDowall, 1994). Over the last ≈30 generations, evidence suggests that detectable population structure has arisen among these NZ populations (Kinnison et al., 2011). As recently outlined by Symonds et al. (2018), the two largest producers, The New Zealand King Salmon Co., Ltd., (NZKS) and Sanford Ltd., produce ∼100–120 families p.a. Initially, NZKS families were reared to tagging size in individual tanks and fish were subsequently tracked using RFID tags inserted into their body cavity at approximately 10 g live mass. Since 2013, communal family rearing has been incorporated for at least some families from incubation onward, with the use of DNA-based parentage analysis (microsatellite markers now transitioned to SNP parentage markers) to identify families at harvest, or prior to spawning. Sanford has also successfully utilized this early pooling strategy using microsatellite DNA markers, now transitioned to GBS.

### Breeding Objectives and Selection Strategies

The initial traits to be selected to meet the industry breeding objectives of increased growth and year-round premium product quality, were weight at harvest (sea pen growth performance), sexual maturation and filet color. By 1997, the first genetic parameters for harvest traits were estimated and included harvest weight, filet color, tail fade (color variation in the tail region of the filet), body fat, filet texture and gaping (slits between the muscle blocks) and 2 year sexual maturation (Jopson et al., 2000; Symonds et al., 2000). Moderate to high heritabilities have been observed for most traits measured. For example, Sanford have found heritabilities in the ranges 0.30 – 0.48 (SE 0.05–0.08) for 2 year-old weight, 0.20–0.84 (SE 0.05–0.09) for color traits and 0.04–0.18 (SE 0.02–0.05) for flesh quality traits for the yearly cohorts of around 1,500 family evaluation fish from 100 to 120 families. With such heritabilities, selection strategies were developed to improve genetic gain. Although positive genetic and phenotypic correlations have been found between color and harvest weight, a positive relationship has also been found between harvest weight and body fat, requiring establishment of a selection index to avoid the potential for increased fat content.

Indices for selection of parents maximized growth rate while maintaining quality and minimizing losses from early maturity. For some breeding programs, selection for body conformation, improved performance and survival over summer are also included. Early maturation at 2 years is no longer a focus as it is controlled mainly through husbandry and the use of lights in the sea pens. Additional traits have been analyzed in the last 6 years including spinal curvature using digital X-radiography (Munday et al., 2016) and feed conversion efficiency (FCE) (Walker et al., 2012). Spinal curvature can reduce size at harvest (Perrott et al., 2018) and result in economic losses due to product down grading. These additional traits have low to moderate heritability, but spinal curvature has been incorporated as a trait in the breeding strategy for one company.

## Greenshell Mussels^TM^

Greenshell^TM^ mussel is the trade-marked name of farmed green-lipped mussels (*P. canaliculus*). A local delicacy for centuries, this endemic species is widely distributed along the coasts of the three main islands of NZ (Powell, 1979). These meaty shellfish are also known locally by the Māori name kūtai and are known globally for their large, colorful shell, affordability, and great eating experience with a good source of protein, low in fat and calories. Furthermore, GSM are a good source of omega-3 fatty acids and minerals (selenium, iodine and iron) essential for good health ^3^.

Farming of GSM (grown to market size on suspended ropes) commenced in the 1970’s to better manage the production of this species which in turn results in better quality than those harvested from the wild. Today, following years of innovation, the industry produces 84,000 to 104,000 metric tons p.a. and is the largest aquaculture industry in NZ based on tonnage and export value. Marketed primarily in the form of frozen on the half shell, the industry exports to over 70 countries^4^.

Although the industry still relies predominantly on wild-caught juveniles (spat), a Primary Growth Partnership (PGP) program operated by SPATNZ is developing commercial scale hatchery protocols. This has enabled the production of selectively bred GSM spat for commercial production where previously farming wild spat precluded genetic improvement. This hatchery is designed to supply ∼30% of current NZ production. The wild spat that supports the industry is sourced from two main areas; attached to seaweed washed onto Ninety Mile Beach (North Island) or collected using specially designed ropes in the northern part of the South Island. However, these spat supplies are both limited and unpredictable, and the retention of spat on seaweed after translocation to mussel farms is typically less than 5 % (Camara and Symonds, 2014). Hatchery production and selective breeding will drive genetic and economic gain, to grow this valuable aquaculture industry.

### Breeding Objectives and Selection Strategies

During the early 2000’s, a novel rearing system for producing large numbers of bivalve families was developed (Ragg et al., 2010) to overcome the issue of high mortality during larval development, a common challenge for shellfish hatcheries. Following single-family tank rearing in this system, rope attachment and deployment in sea-based farms, individuals are grown until they are ∼40–50 mm in shell length and family identification numbers engraved on the shell. This enables commercial evaluation trials to be conducted by mixing these marked animals for re-seeding onto rope “droppers” to minimize common environmental effects in the field. These mixed-family droppers are then harvested at a standard size of ∼95 mm shell length and destructively sampled to provide family specific measurements as part of the selection process. Separate, single-family droppers are also held as broodstock.

Breeding has focused on producing attractive, fast growing, resilient and high-yielding mussels suited to the predominant half-shell product. Reviewed in Camara and Symonds (2014) and Hess et al. (2018), the heritabilities for key traits are moderate to high with few strong adverse genetic correlations between traits. Approximately 125,000 mussels from 550 families produced over 9 cohorts have been assessed, yielding heritabilities of 0.30 (SE = 0.077) to 0.43 (SE = 0.069) for shell dimensions and 0.17 (SE = 0.064) for meat weight (Camara and Symonds, 2014). As well as selection for established commercial traits, the program continues to evaluate additional traits to assess their future potential for selection and to understand the physiological changes in the mussels in response to breeding (Ibarrola et al., 2017). For example, live air shipment of GSM to high-value markets represents an opportunity for the industry. Emersion tolerance (survival in air) varies among families (Powell et al., 2017) and is moderately heritable (Camara and Symonds, 2014). Preliminary analysis also suggests different families have varying resilience to ocean acidification during the fragile early life stages (Ragg, unpublished data).

## Genomic Solutions for the NZ Aquaculture Industry

The current selection methods utilized by the industries discussed are pedigree-based mixed-model BLUP (best linear unbiased prediction) to estimate genetic parameters and breeding values (EBVs) using quantitative measurements on individuals (recently reviewed in Symonds et al., 2018). These BLUP selections rely on either recorded pedigree and/or pedigree established/verified by DNA parentage methods. The two large salmon companies have recently transitioned from microsatellite parentage panels to use of SNP technology, either in the form of low-plex SNP parentage panels or restriction enzyme based GBS (RE-GBS) technology that enables thousands of SNP markers to be interrogated and utilized. Both of these SNP genotyping methods have the potential to be cost competitive in selective breeding programs. An advantage of RE-GBS to that of fixed genotyping platforms (e.g., array based, targeted GBS, amplicon sequencing) is the ability for simultaneous SNP discovery and genotyping (Elshire et al., 2011; Dodds et al., 2015; Ashby et al., 2018; Kristjánsson et al., 2018; Palaiokostas et al., 2018). Further, GBS technology gives flexibility in genotyping a large number of training and prediction individuals for genomic selection (Gorjanc et al., 2015, 2017). GBS is of particular use for emerging and/or minor industries, where fixed array-based methods can have high set-up costs and subsequent on-going genotyping costs that may be cost prohibitive in aquaculture breeding schemes. Furthermore, oligo-based genotyping can be problematic in highly polymorphic species and populations with high genetic diversity, such as molluscs.

Recently, SPATNZ has begun investigating the potential for RE-GBS to complement the GSM selective breeding program (Ashby et al., 2018; Hess et al., 2018). Through the construction of genomic relationship matrices (GRM) utilizing ∼100,000 SNP markers, RE-GBS has been employed for parentage assignment and to derive genomic breeding values (Ashby et al., 2018; Dodds et al., 2018). Preliminary results comparing phenotypically derived EBVs with genomic breeding values (calculated by masking the phenotypic data in a validation subset of genotyped animals) indicated the potential for improved accuracy for shell length compared to that of pedigree based breeding values from 0.22 ± 0.25 to 0.46 ± 0.14 (Ashby et al., 2018). The NZ salmon industry has also recently employed RE-GBS to establish pedigree and make use of the GRM to inform mating decisions and establish the genetic diversity of their broodstock.

### GBS in NZ King Salmon

NZ king salmon aquaculture companies are employing RE-GBS for a range of applications; from pedigree assignment (allowing calculation of EBVs), positioning for use in genomic selection whereby trait and genomic information are simultaneously collected, to assessing the genetic diversity of broodstock that have never been DNA profiled previously. All of these applications are making use of the KGD method from Dodds et al. (2015) that establishes kinship from GBS data while taking read depth into account, thereby enabling a more cost-effective DNA profiling tool that assesses between 0.8 and 8% of the genome depending on enzyme choice. This low-depth sequencing approach coupled with high-throughput, high quality DNA extraction methods (Clarke et al., 2014) and reduced sequencing costs has the potential to provide information such as genetic diversity indices to genomic selection, all for a similar cost to that of low-plex DNA parentage panels. This is particularly important for many smaller companies where individual pedigree information is not available. The following example is given to highlight how RE-GBS can be utilized to inform spawning decisions, assess inbreeding and determine effective population size of a breeding population.

#### GBS Preparation and Processing

The RE-GBS was performed on a subset of males and females from 2 year classes (2014; 2015; *n* = 469) as described in Dodds et al. (2015) using PstI/MspI restriction enzymes and library selection size of 193–318 bp. Following data processing^5^^,^^6^, the variants, together with the recorded allelic depth, were utilized for the KGD software^7^ to establish relatedness within (self) and between the samples. Using the following filtering parameters applied to the data (sample depth > 0.3, observed Hardy-Weinberg disequilibrium > -0.5), ∼46 K SNPs were observed. Processing 120–380 samples/lane (1 × 101 nt sequencing read and 27 Gb data/lane), the mean sample depth and call rate ranged from 2.17 to 4.10 and 0.66 to 0.80, respectively, with a mean self-relatedness of 0.98 observed.

#### Assessing the Broodstock

Utilizing allelic data, levels of genetic variation within the broodstock were determined by three different estimates of diversity: number of variable SNPs detected within the group, Minor Allele Frequency (MAF), and effective population size, N_e_. The effective population size is the size of an idealized population (random mating between and within sexes including selfing) that would give the same rate of increase in inbreeding as the population under study. The N_e_ estimation was based on the *r*^2^ measure of linkage disequilibrium between SNPs on different chromosomes (Hill, 1981; Barbato et al., 2015), with *r*^2^ estimated taking read depth into account and allowing for the possibility of sequencing errors (Bilton et al., 2018). The first SNP on each chromosome was used to create a subset of unlinked SNPs as required to estimate N_e_ for the current generation. Median values of *r*^2^ were used instead of means, to guard against mis-assignments to chromosomes (which were based on an assembly of the rainbow trout). Sample size corrections were not used, as medians of ratios were used instead of means.

**FIGURE 1:**
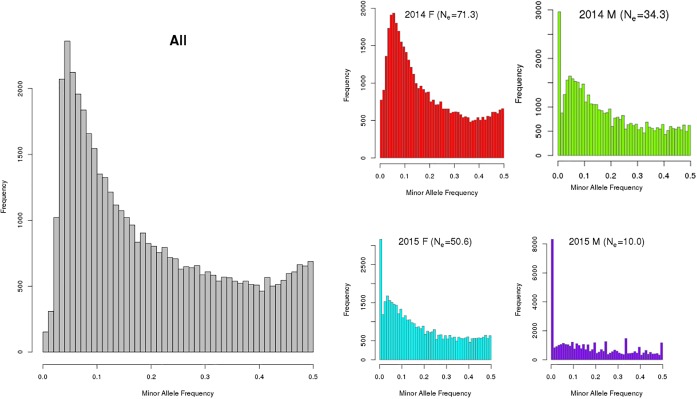
Minor allele frequency distribution of combined (All) and by year class [2014 and 2015; females (F) and males (M) for (king salmon the value in brackets in the individual graph title is the estimated Ne)]. Note: The *y*-axis scale varies between these plots and a MAF of zero indicates a SNP is detected in at least one of the 4 groups but not present (i.e., had only one allele) in the specific group being analyzed. The number of SNPs (MAF > 0) observed were 46,007 and 43,838 for the 2014 female (*n* = 220) and male (*n* = 55) year class, respectively, and 44,114, and 38,323 for 2015 female (*n* = 159) and male (*n* = 35) year class, respectively. Note that only a small sub-set of the 2015 year class males had been sampled at the time this analysis was completed and were included for comparison.

The number of informative SNPs (>0) ranged from ∼38 to ∼46 K, with the MAF distributions from combined and per group shown in Figure 1. In a population with low N_e_, rare alleles [e.g., MAF < 5% (0.00)] are easily lost (resulting in a MAF of zero) so that many of the remaining SNP are represented as having common alleles [e.g., MAF > 20% (0.20)]. In a frequency distribution (see Figure 1), a higher proportion of individuals with less common or rare alleles produces a distribution that is skewed left (ignoring MAF = 0 data).

The diversity measures are consistent in that the number of polymorphic SNPs increases as the proportion of polymorphic SNPs with MAF > 0.2 decreases and as N_e_ increases. Males from each year class have the least diversity (lower N_e_, lower number of polymorphic SNPs, flat MAF distribution for the polymorphic SNPs) with the 2014 females exhibiting the most diversity. In males the estimated effective population sizes are lower than desirable whereby a total N_e_ of 50 per year class is recommended in breeding programs to avoid loss of genetic variation and restrict inbreeding (Franklin, 1980; Soulé, 1980). However, more 2015 males will be genotyped in the future from a different hatchery source and the N_e_ is expected to increase.

**FIGURE 2:**
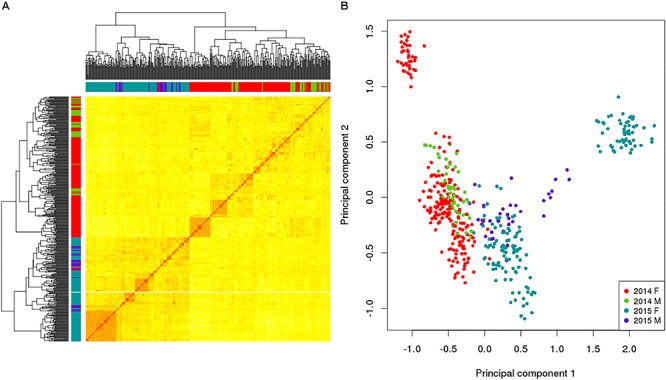
Genomic relationship matrix **(A)** and principal component analysis **(B)** for a subset of king salmon males and females from the 2014 and 2015 year classes; self-relatedness on the diagonal and relatedness between individuals on the off diagonal. The colors in the sidebars of the GRM and the dots on the PCA refer to the broodstock group origin of the individual (Red 2014 Female, Green 2014 Males, Blue 2015 Female and Purple 2015 Male). For the GRM the darker (and redder) the color in the matrix, the more related the individuals. The PCA explains 23.6% in the first component and 9.5% in the second.

In addition to utilizing the allelic data to assess the levels of genetic variation within the broodstock, the allelic sampling from the GBS data was used to calculate the relatedness between all the possible combinations of the 469 broodstock using the KGD method (Dodds et al., 2015) and is depicted in the GRM heatmap (Figure 2A). This displays the self-relatedness on the diagonal and relatedness between individuals on the off diagonal with each group color-coded on the axis. The heatmap helps understand the relationships between groups (color coded) and individuals. Being able to estimate relatedness allows estimation of breeding values and construction of mating designs to control inbreeding.

#### Genetic Diversity Summary

This example of utilizing RE-GBS coupled with KGD analysis indicates that there can be low levels of genetic variation within some king salmon broodstock groups based on number of SNPs observed, their MAF distribution, and the low N_e_. Distinct groups by year class were also detected in the data subset (Figure 2B). The 2014 and 2015 broodstock are not closely related and the PCA found 23.6% diversity explained by the first component which differentiated the year classes. These results could be utilized to implement a broodstock genetic management plan to address levels of genetic diversity observed in the 2 year classes, implying that ongoing GBS of broodstock is advisable to ensure sufficient numbers of minimally related broodstock are used as parents and for maintenance of genetic diversity. Furthermore, sourcing additional broodstock from other populations is also an option and could provide additional variation, preferably with prior genotyping to assess relatedness to current broodstock and target the least related individuals. This would be undertaken if the increased diversity was thought to outweigh any possible decrease in genetic merit. The example provided is based on a subset of data from 2 year classes and does not reflect the overall genetic diversity of the NZ king salmon industry, but was utilized to highlight the utility of RE-GBS for estimating the genetic diversity of broodstock.

## Future Opportunities

The above example highlights the utility of RE-GBS to provide a genetic diversity summary, over and above DNA parentage for a cost similar to many oligonucleotide low-plex SNP methods for parentage assignment. The full potential will be realized when “DNA parentage” genotyping is replaced with genotyping methods that offer greater efficacy, such as RE-GBS. This simultaneous SNP discovery and genotyping platform enables genome wide analysis and development of genomic selection in aquaculture systems without set-up costs associated with targeted GBS and fixed SNP array technologies, where on-going genotyping costs can be high, particularly in smaller industries where large volume discounts may not be achieved. This paper has shown that selective breeding of NZ aquaculture species is well established and the benefits from breeding have been clearly demonstrated. Within the next decade we expect those benefits to grow through the application of modern genomic techniques and RE-GBS provides a cost-effective method, not only within the established family programs, but also for smaller companies who previously have had no genetic management.

## Author Contributions

All authors contributed to the development of the aquaculture breeding programs in NZ, on which this perspective is based and provided information that was included in this paper. JS, SC, and KD wrote the first draft of the manuscript with input from SW, NK, and RR. JS, SW, BB, and DS organized the sampling of the salmon broodstock. RR, NK, JS, SC, and KD provided the genomics information. SC and KD completed the salmon GBS and analysis. All authors contributed to manuscript revision, read and approved the submitted version.

## Conflict of Interest Statement

BB, DS, PB, MP, RR, and MT are employed, or sub-contracted directly, by commercial aquaculture companies in New Zealand. The remaining authors declare that the research was conducted in the absence of any commercial or financial relationships that could be construed as a potential conflict of interest.

## Footnotes

1 www.aquaculture.org.nz

2 www.salmon.org.nz/

3 www.foodcomposition.co.nz

4 www.aquaculture.org.nz

5 https://github.com/AgResearch/DECONVQC

6 http://www.maizegenetics.net/tassel

7 https://github.com/AgResearch/KGD
